# Perception and valuations of community-based education and service by alumni at Makerere University College of Health Sciences

**DOI:** 10.1186/1472-698X-11-S1-S5

**Published:** 2011-03-09

**Authors:** Andrew Mwanika, Isaac Okullo, Dan K Kaye, Wilson Muhwezi, Lynn Atuyambe, Rose C Nabirye, Sara Groves, Scovia Mbalinda, Gilbert Burnham, Larry W Chang, Hussein Oria, Nelson Sewankambo

**Affiliations:** 1 College of Health Sciences, Makerere University, Kampala, Uganda; 2 Johns Hopkins School of Medicine, Baltimore, Maryland 21205, USA; 3 Johns Hopkins School of Nursing, Baltimore, Maryland 21205, USA; 4 Johns Hopkins Bloomberg School of Public Health, Baltimore, Maryland 21205, USA

## Abstract

**Background:**

Training of health professionals can be deliberately structured to enhance rural recruitment by exposing the trainees to the realities of rural life and practice through Community-Based Education and Service (COBE) programs. Few studies have surveyed the alumni of these programs to establish their post-university views and whether the positive impact of COBE programs endures into the post-university life. This study surveyed the alumni of COBE at Makerere to obtain their perceptions of the management and administration of COBE and whether COBE had helped develop their confidence as health workers, competence in primary health care and willingness and ability to work in rural communities.

**Objectives:**

• To assess the efficiency of the management and administration of COBES.

• To obtain the views of the impact of COBES on its alumni.

**Methods:**

A mixed qualitative and quantitative study was conducted using focus group discussions (FGD) and a telephone administered questionnaire. From a total of 300 COBES alumni 150 were contacted. Twenty four Alumni (13 females and 11 males) were purposefully selected by discipline, gender and place of work, and invited for the focus group discussion. The discussions were transcribed and analyzed using a manifest content analysis table. The thematic issues from the FGDs were used to develop a structured questionnaire which was administered by telephone by the authors. The data were entered into Microsoft excel template and exported to Stata for analysis. The findings of the telephone survey were used to cross-match the views expressed during the focus group discussions.

**Results:**

The alumni almost unanimously agree that the initial three years of COBES were very successful in terms of administration and coordination. COBES was credited for contributing to development of confidence as health workers, team work, communication skills, competence in primary health care and willingness to work in rural areas. The COBES alumni also identified various challenges associated with administration and coordination of COBES at Makerere.

**Conclusions:**

This study has established that the positive impact of COBES endures with the alumni of the program. Health planners should take advantage of the impact of COBES and provide it with more support.

## Background

Within the context of the human resources for a health crisis, one of the most complex challenges facing health policy makers and planners is to ensure that people living in rural and remote areas have equitable access to trained health workers [1]. Training of health professionals can deliberately be designed to enhance rural recruitment and retention by exposing the trainees to the realities of rural life and practice [2,3]. The rural core clinical rotation program in the University of Queensland for instance was initiated in 1999 with the aim of enhancing opportunities for rural recruitment by exposing the students to the real life issues faced by the rural and remote communities [4]. In Mali an in-service training program was specifically designed to socialize doctors into their rural role, and help them internalize norms, values and practical attitudes that are characteristic of rural practice [5].

Several health professionals training institutions in Uganda and elsewhere have adopted community-based education and service [COBES] as an innovative approach that is likely to produce health workers who are equipped and willing to work in rural areas [6-8]. The COBES experience generates emotional consequences which challenge students’ ideals and values and contributes to personal development, a sense of maturity and development of civic responsibility [4]. It also produces health workers with locally relevant skills, who are community service oriented [9].

Makerere University College of Health Sciences (MakCHS) underwent an extensive curricula evaluation and change in 2003. One of the key initiatives undertaken was the development and integration of COBES into the health sciences clinical degree programs: medicine, dentistry, nursing, radiography, and pharmacy [10]. Students in these programs now participate in COBES rotations every year, rotating for over five weeks, at over forty community-based sites. During the first three years after initiation, the COBES activities were financed largely through donor funds from Rockefeller Foundation supplemented by a World Bank loan to Uganda for use by Makerere University. The above funds were jointly used to support innovations at Makerere University of which COBES was an outstanding example of such an innovation. After the initial funding, Makerere University sustained the COBES program from the government subvention to Makerere and internally generated funds. Preliminary findings in a study of the administration and management of COBES in various health training institutions including MakCHS in Uganda indicate that funding of COBES is inadequate and has impacted the quality of supervision, students’ welfare and site activities [11]. This is especially true for Makerere with the completion of the World Bank and Rockefeller project funding.

The objectives of COBES at MakCHS are to prepare health professionals for rural practice and primary health care, integrate priority national health programs into the undergraduate training, bridge the manpower gaps during training through service learning, and to shift the focus of health intervention from the facility to the communities and households. Others are to train health workers who are community leaders, community researchers, service providers, change agents and team players. [COBES Guidelines Booklet, unpublished].

The success and sustainability of COBES depends in part on its administration and coordination and on the perceptions and value attached to it by its clients. COBES clients can be differentiated into internal clients and external one. The internal clients include students, faculty, site tutors (preceptors), and alumni. While the external include the district staff and communities where COBES activities are located and occur. The perceptions and valuations of faculty, students and site tutors were surveyed and reported by the authors in a separate paper [12]. The views of the external clients are also reported in a study of the communities where the interventions occurred [13].

Since adopting COBES, MakCHS has graduated two cohorts of nurses and pharmacists and one cohort of medical doctors and dentists. As few studies have reported on the impact of COBES on alumni activity, we conducted a survey of MakCHS COBES alumni on their perceptions of the management and administration of COBES, and whether they felt COBES had helped to develop their confidence as health workers, competence in primary health care, and willingness and ability to work in rural communities.

## Methods

The study was both qualitative and quantitative using focus group discussions and a structured questionnaire for the qualitative and quantitative findings respectively.

### Study setting and participants

The study was conducted at MakCHS from 1^st^ May to 10^th^ July 2010. In May 2010 the College had graduated a total of 300 students who had started in or after 2004, with two cohorts of nurses and pharmacists (2008 and 2009), and one cohort of doctors and dentists (2009). Participants for both the focus group discussions and the telephone survey were drawn from these 300 COBES alumni with representation from all the disciplines and both gender. The focus group discussions were held over one morning lasting between 2-3 hours. The telephone survey was conducted over a period of four weeks.

### Study sample for the FGDs

From a total of 300 COBES alumni, 150 were contacted through snowball sampling using telephone calls. During the calls, the alumni were asked to join the study and once they accepted to provide information on both their discipline and place of work, specifying whether they were urban or rural based, and whether they worked in a public or private facility. They were invited to participate in the study as data sources. All areas gazetted as district headquarters, cities, towns and all townships with more than 1000 people were regarded as urban [Uganda Bureau of Statistics compendium 2001 unpublished]

Twenty four participants, (13 females and 11 males) were purposefully selected from the list of 150 to represent all disciplines, both genders and a variety of work places. Those selected were invited to participate in a focus group discussion (FGD). The plan was to form four discussion groups and therefore six alumni were invited for each group. In the end, only three discussion groups were formed because there were not enough rural private practice alumni to form a separate group. The alumni working for NGOs or faith based facilities in the rural areas were classified as rural based private practitioners. Group one had six alumni (four males and two females) who were in private practice (both urban and rural), group two had eight alumni (six males and two females) working in urban based public facilities, while group three had six alumni (three males and three females) working in rural based public facilities.

### Study sample for the telephone survey

The remaining list of 126 alumni with their telephone contacts was systematically divided and distributed among six members of the research team. Each member received about 22 alumni to interview. The survey tool for the telephone interviews was structured with mostly close ended yes/no questions. The interviews on the average lasted 20-25 minutes. Data were successfully obtained from 90 out of the 126 contacts, giving a response rate of 71%.

### Data collection and analysis

The FGD were facilitated by experienced staff using guides that had been developed and piloted the previous two weeks by the authors. Each group also had a scribe who took notes and recorded the proceedings using a tape recorder. The guide was developed to focus participants’ views regarding administrative and management issues that included: pre-placement preparations, communication, student welfare and supervision. The guide was also designed to explore the relevance of COBES and its ability to inspire confidence, develop professionalism, and willingness of trainees to work in rural communities.

The discussions were transcribed by the scribes and analyzed using a manifest content analysis table showing themes, views and the number of responses each issue elicited from each theme by the 3 group members. The thematic issues from the FGDS were used to develop a structured questionnaire, which had 24 close ended questions and six open ended ones. The authors then administered this tool with a telephone survey. The data from the telephone survey were entered into Microsoft excel template and exported to Stata for analysis. The findings of the telephone survey were used to cross-match the views expressed during the focus group discussions.

### Ethical considerations

The research was approved by the Institutional Review Boards of the College of Health Sciences Makerere University and Uganda National Council of Science and Technology. Johns Hopkins School of Public Health IRB also assessed the project and determined that no additional approval was required. All participants gave informed consent. They also received a guarantee that the information they provided was confidential and there would be no personal identifiers in any documents or subsequent reports.

## Results

The views from the Focus Group Discussions are presented in form of thematic narrative while the qualitative results from the telephone survey are presented in two tables.

### Qualitative results

The findings from the focus group discussions are presented under major themes namely: management and coordination of COBES educational program, COBES’s contribution to development of confidence and competence as health workers, professionalism and teamwork, willingness to work in rural health facilities and practice of primary health care. In general there were no significant differences in view points between the three groups.

### Management and coordination of COBES educational program

The findings under the above theme are presented under the sub-themes of pre-placement preparation of students, communication, student supervision, and welfare.

#### Pre-placement student preparation

All the groups strongly expressed the view that pre-placement preparation characterized by overview lectures, workshops, briefings and communication to the COBES sites were good and well coordinated during the initial two years of the program. However they felt that by the third year, the coordination and pre-placement preparation had became lax and less stringent. This is born out by statements like: *“My best COBES was in first year because it was well organised and we were well prepared*, *we had sufficient funds and all the reading materials*, *the sites too were ready for us.”* (FGD, Alumni)

Another discussant said, “I *concur that COBES in the first year was really perfect, we were told what we where going to do, the site tutors had their guides and knew we were coming.*” (FGD, Alumni)

#### Communication

Communication was discussed in terms of communication between MakCHS and COBES sites on one hand and between students and site supervisors and feedback to the community by students on the other hand.

Communication between the College and the COBES sites was cited as good especially in the initial years. *“Communication between site tutors and the faculty, in the first year of COBES was good, I remember our site tutor even used to call the College incase of anything unclear.”* (FG, Alumni)

Students were also encouraged to communicate with the sites before departure and some did. *“Yes, we could communicate to the site and they could get us a house within our budget. Then some of us could go with mattresses so by the time we got there, we are well prepared and faced no problem with accommodation.”* (FG, Alumni)

As far as feedback is concerned, most discussants agreed that they gave feedback to the districts in form of reports. However they also agreed that direct meetings with the community and other stakeholders for feedback did not take place and yet would have been useful. *“Ultimately if we are to improve this system we need to bring all three stakeholders*, *students*, *the faculty organizers*, *districts and the community to a feedback meeting.”* (FG, Alumni)

#### Student supervision

There were two aspects to student supervision that were discussed. The first was supervision by faculty, referred to as site supervisors, and the second is the supervision by the preceptors known as site tutors. All the groups were unanimous that initially the site supervisors were very committed and would make two visits per placement which was very useful in helping the students. Beginning with the third year, the frequency of visits was reduced and the quality of visits was less satisfactory. *“In first year they came twice*, *attended tutorials with us and spent the whole morning*, *checked our log books to see whether we are logging in the right information*, *so their participation was adequate. However they started coming less frequently in the last year.”* (FG, Alumni)

#### On-site supervision

The majority view that emerged regarding the site tutors was that they were always committed and when not available delegated the supervision to other facility staff.

*“Site supervision was very good because like it was said they delegate – for whatever activity we were doing to whoever was in charge of that particular activity” “One thing you can note from the site tutors is that there is absolute willingness to participate in COBES.”* (FG, Alumni)

#### Student welfare

This was discussed in terms of adequacy and timeliness of the allowances paid to facilitate the students’ stay at the COBES sites and availability of accommodation. The issue of students’ welfare followed the same pattern, as seen above. Initially, the funds were perceived to have been adequate and were provided on time for the students to depart to their sites. Most of the discussants in all the groups said that they either found free accommodation arranged at the COBES sites and if not present, could afford to rent good accommodation with the allowance they received.

*“Again in the third year, not only were the funds for allowances reduced but they never came on time.”* (FG, Alumni)

*“In my first year facilitation was really nice and I even had excess.” In 3^rd^ year the funds were little and came late.”* (FG, Alumni)

*“Funds were provided on time in first year and we were able to get good accommodation.”* (FG, Alumni)

### Does COBES contribute to development of confidence as a health worker?

All the three groups expressed agreement that COBES had contributed to their development of confidence as health workers. Confidence building and development of competence were seen in relation to both clinical and non-clinical areas like public speaking.

*“COBES helped develop my confidence. There are so many things I leant from COBES that I would probably not have had a chance to learn here, at the Medical school. For example under supervision I learnt how to administer injections during my first year COBES rotations.”* (FG, Alumni)

*“I learnt about medicines because we got a chance to go into the pharmacy, and be taught how to dispense medicines– okay those may be minor things but when you start to practice you realize that you need them also.”* (FG, Alumni)

*“When you are in the community during COBES you have a chance to learn and do a lot of things. Now that we are interns in the surgical unit we run it as registered doctors do. – You don’t have to call anyone to canulate; for by the time you get there you are completely confident because you have had 4 years during COBES of practicing to do something and you are okay with it. So COBES provides a more convenient and conducive environment for learning.”* (FG, Alumni)

*“COBES also opened a way for us to enter into different homes, communities and offices and this has helped us gain confidence of our service delivery. It also taught us how to work as a team.”* (FG, Alumni)

### Does COBES contribute to development of communication skills?

The majority view was that COBES did contribute to development of competence in communication skills.

*“In my fourth year of COBES we had to speak to the public about prevention of dental cavities. I enjoyed this a lot and I am glad I acquired this skill, because even where I am now I always go out to the communities and teach prevention.”* (FG, Alumni)

*“COBES gave me the confidence which I think I would not have got if I had stayed in medical school. It taught me how to talk to strangers. It taught me about public speaking, how to communicate to masses of people.”* (FG, Alumni)

### Does COBES contribute to development of community health skills?

There was general agreement that COBES had contributed to development of community health skills. And this was expressed as follows:

*“We learned the link between sanitation and health and why in real life water springs are protected and water taps are provided. These things cannot be properly appreciated in Medical schools to learn these things which are key to good community health we have to go to the communities and COBES provided us with the opportunity to this.”* (FG, Alumni)

*“COBES was very instrumental in teaching me family dynamics and their role in health.”* (FG, Alumni)

*“COBES helped me appreciate health as a whole. You know when you join Medical school, you think of say becoming a pharmacist and concentrating on knowing medicines and being an expert in that. However COBES gives you a chance to appreciate those other attributes and for instance also learn how to counsel and speak to people. Because those are skills you also need as a pharmacist.”* (FG, Alumni)

*“As a nurse and I learned during COBES that when implementing public health or community health issues it is not just about telling people what to do. It is much better for one to get involved and do it then the people join in”. In fact it is also a form of communication.”* (FG, Alumni)

### Does COBES contribute to development of willingness to work in rural areas?

The discussants agreed that COBES had indeed contributed to their willingness to work in rural areas. But they also strongly called for a need to provide equipment and other infrastructure in these areas.

*“I definitely attribute my willingness to work in rural areas to COBES. The beauty of working in a rural area is people appreciate that you are around and everybody tries to get something good out of you.”* (FG, Alumni)

*“COBES opened up the other angle that you can go out and become a better medical worker and yes COBES prepared me to work in a rural area too.”* (FG, Alumni)

“COBES played a big role in our exposure to real-life rural settings, I have got used to rural areas and its exposure, and I don’t mind working there.”

*“With the COBES experience and background I can adapt and work almost anywhere including in rural areas.”* (FG, Alumni)

There were however calls for provision of appropriate infrastructure in the rural areas to facilitate work in these areas.

*“I wouldn’t mind working there if structures and patient care were improved; there is a shortage of modern equipment to perform even the basic procedures on patients.”* (FG, Alumni)

### Does COBES contribute to development of professionalism?

The focus groups presented aspects of professionalism that they felt COBES had helped develop in them including team work, civic responsibility, personal development, and cultural sensitivity. Their views are represented by the following examples:

*“Team work was emphasized during COBES because as health workers teamwork is vital and needed in order to properly provide services to society”*. (FG, Alumni)

*“COBES had a positive impact on my professional conduct because I got to know the importance of our role as doctors to society because you go out and you see much and learn what has to be done to better and improve the health of the local people. So COBES helped me in that way.”* (FG, Alumni)

*“We learned to work in the rural areas, without even thinking of money as most rural people don’t really have it and can not afford to pay for services. This helped us develop responsibility to the people.”* (FG, Alumni)

*“Sometimes we were asked questions that we were not in position to answer immediately. But we would look for answers through reading which helped improve on our professional knowledge”*. (FG, Alumni)

*“It helped me to develop myself because in my 2^nd^ year, 3^rd^ and 4^th^ year December holidays we kept on going to hospitals where if they gave us food and accommodation. We were useful but at the same time developed ourselves.”* (FG, Alumni)

*“COBES gave me confidence to work in different communities, with different cultures. And it also gave me the confidence to net-work with colleagues and develop professional-interpersonal skills. My site tutor was clinical biased and I leant a lot from him which I am now using. COBES helped a lot and that’s why I am upcountry I got used to working and living upcountry.”* (FG, Alumni)

### Does COBES develop competence in Primary health care skills?

Strong linkages were made between the COBES experience and development of Primary health care skills.

*“Cobes helped me learn to focus on disease prevention as well other than just the curative approach. So we spent time educating people on disease prevention instead of waiting for them to come for treatment.”* (FG, Alumni)

*“I would say COBES taught us Primary health care skills for example, in designing community health projects we learned to factor in sustainability of the project, through community acceptability – because we wouldn’t like to design a project which would cease as soon as we exit the community. We wouldn’t have had the opportunity to do this anywhere else except in COBES.”* (FG, Alumni)

### Quantitative findings

Out of the 90 alumni that participated in the telephone survey, 55 (61.1%) were males and 35 (38.9%) were females. Of the respondents 37 (41.1%) had completed COBES placements in urban health facilities and 53 (58.9%) in rural health facilities. An urban site was defined as one located within the officially gazetted administrative authority of a city council or other municipality or town council. Rural sites were those located outside the urban environs as defined above.

The results of the telephone survey are presented below in two tables. Table 1 contains responses to questions around variables associated with the administration and coordination of COBES at MakCHS. Table 2 shows views of the impact of COBES on the alumni with respect to development of confidence, professionalism, sense of responsibility, willingness to work in rural areas and primary health care skills.

**Table 1:** Alumni views on the administration and coordination of COBES

VARIABLES	**RESPONSE** [frequency & percentages]	
	**YES**	**NO**

Do the overview lectures give you information about the COBES sites?	51 (56.67%)	39 (43.33%)
Are briefing meetings held before departure for COBES?	83 (92.22%)	7 (7.78%)
Is it necessary to have briefings before departure for COBES?	88 (97.78%)	2 (2.22%)
Was your site tutor prepared for you?	68 (75.56%)	22 (24.44%)
Do you feel it’s beneficial to give feedback?	90 (100%)	0 (0%)
Did your faculty supervisor come at appropriate times?	50 (55.56%)	40 (4.44%)
Was your site tutor helpful?	85 (94.44%)	5 (5.56%)
In cases where your site tutors delegated, was it helpful?	61 (67.78)	27 (30%) 2 (2.22%) no response
Were the faculty supervisors familiar with the students' projects?	69 (76.67%)	21 (23.33%)
Was your faculty supervisor helpful?	60 (66.67%)	30 (33.33%)
Comment on adequacy of communication between students and faculty supervisors.	Adequate 33 (36.67%)	Not Adequate 57 (63.33%)
Comment on adequacy of communication between students and site tutors.	Adequate 78 (86.67%)	Not Adequate 12 (13.36%)

**Table 2:** Alumni views on the impact of COBES

VARIABLES	**RESPONSES** [frequency & percentages]	
	YES	NO

Did COBES help you develop confidence to work as a health worker?	76 (84.44%)	14 (15.56%)
Did COBES affect you willingness to work in rural stations?	75 (83.33%)	15 (16.67%)
Are you willing to work in rural areas?	78 (86.67%)	12 (13.33%)
Has COBES contributed to the development of your professional conduct as a health worker?	79 (87.78%)	11 (12.22%)
Has COBES helped you develop sense of responsibility towards Community?	79 (87.78%)	11 (12.22%)
Did COBES prepare you for primary health care?	89 (98.89%)	1 (1.11%)

## Discussions

The alumni almost unanimously agreed that the initial three years of COBES were very successful in terms of administration and coordination despite absence of an established COBES coordination department. They cite pre-placement preparation in form of overview lectures, workshops, and the briefing meetings as having been well organised and useful in preparing them for their COBES experiences.

Smith [14] found that poor student preparation especially in terms of “uninformed expectations” and lack of on-site supervision are some of the barriers influencing the ability of junior doctors to practice competently and confidently in rural and remote areas. COBES’s success in building the confidence and willingness of the alumni to work in rural areas can be attributed, in part, to the pre-placement preparation of the students which provided information about rural life and practice, and helped create realistic expectations.

The other strong point raised by the alumni is the quality of on-site supervision which they said was reliable and always available, provided either by the site tutor (preceptor) or by a delegated staff of the facility. A study of the perceptions and valuations of continuing students at MakCHS demonstrated a high value attached to the site tutors by the students because they were readily available, gave constructive feedback, respected students’ judgment, and provided good role models for professional conduct [12].

The COBES alumni attached a premium on COBES accrediting it with contributing to development of confidence as health workers, team work, communication skills, competence in primary health care and willingness to work in rural areas. COBES scores impressive figures as far as preparation of students for rural practice is concerned.

The telephone survey found that 86% of the respondents were willing to work in rural areas, and it was COBES that affected their willingness to do so. A similar proportion of 87% admitted that COBES helped them develop a sense of civic responsibility and community commitment, while 98% believed that COBES had helped to prepare them for primary health care. Willingness to work in rural areas is accompanied by a strong call from the discussants for provision of equipment and other infrastructure in these areas. Indeed the latter are some of the challenges or push factors associated with difficulties to retain health workers particularly in rural areas [15].

Several studies have evaluated the impact of community-based education programs on students and found a positive impact especially in terms of preparing trainees for rural practice [16,17]. This study provides evidence which strongly points to the possibility that the positive impact of COBES endures with the alumni into the post-university years. This information could be of strategic value as far as recruitment and retention of health workers for rural health facilities is concerned. Adoption of Community-Based Education programs, if combined with provision of a good working and living environment in rural areas, could produce what Lehmann [15] calls a “bundle of comprehensive interventions” which are more likely to succeed in attracting and retaining health workers in these areas.

Despite the positive experiences, the COBES Alumni identified various challenges associated with administration and coordination of COBES at Makerere including inadequate communication, infrastructure and access to learning materials. In addition they identified a need for increased faculty supervision, improvements in site and student preparation, and stronger support for students’ welfare. Neil and Taylor [18] found that rural and remote experiences increased students’ interest in returning to rural nursing, but adequate financial support for the students and for their supervision during rural placement is necessary. During key informant interviews, we learned through the program coordinator at Moi University that both the students’ welfare and supervision costs are in-built into the fee structure, which helps ensure availability of dedicated funds for these activities [11].

The alumni, as we have established, place a premium on COBES. The value of COBES depends in part on its management and coordination. The efficient management and coordination of the program as has been testified by the alumni helps prepare the students and creates an environment which promotes positive COBES learning experiences. Negative rural learning experiences which can arise from poor management and coordination can result in the students rejecting rural practice in favour of urban practice

## Conclusions

The alumni of COBES at MakCHS testified that COBES had a successful start, but needed more structural and financial support to ensure its continued success. Both the internal and external clients of COBES placed a high premium on it which is partially responsible for its current success.

This study has established that the positive impact of COBES experience gained during training endures with the alumni of the program. In as much as the human resources are the most critical element in the endeavor to provide health services, their training is equally critical in appropriately preparing them for their roles. One of the key challenges today is to prepare trainees for primary health care and rural practice in order to enhance rural recruitment and retention. Community-based programs have been demonstrated to shape the students’ attitudes and skills towards rural practice. This study has shown that this positive impact of COBES endures with the alumni of the program.

It’s important to invest in more support to Community-Based Education programs and to mainstream them into the training of all cadres of health professionals. At the same time health planners should take advantage of the impact of Community-Based Education programs and work to provide a “bundle of comprehensive interventions” which take into account community-based training and improved work and living conditions in rural areas.

The purpose of this study which was to obtain the perceptions and valuations of the COBES program by Makerere Alumni was achieved. However there is need for a longitudinal tracer study of the alumni which can establish choice, longevity and competence of rural practice by COBES graduates.

## List of abbreviations used

COBES: Community-Based Education and Service; FGD: focus group discussions; MakCHS: Makerere University College of Health Sciences.

## Competing interests

The authors declare that they have no competing interests.

## Authors' contributions

AM, DK, GB were involved in the conception of this paper, data analysis, and drafted the manuscript. IO, WM, LA, RN, SM, HO, NS were involved in the conception of the paper, data collection and qualitative data analysis. SG and LWC were involved in the conception of the paper and drafting the manuscript. NS contributed to the review and writing of the manuscript. All authors have read and approved the manuscript.
